# Hair cortisol level might be indicative for a 3PM approach towards suicide risk assessment in depression: comparative analysis of mentally stable and depressed individuals versus individuals after completing suicide

**DOI:** 10.1007/s13167-022-00296-z

**Published:** 2022-08-30

**Authors:** Alexander Karabatsiakis, Karin de Punder, Juan Salinas-Manrique, Melanie Todt, Detlef E. Dietrich

**Affiliations:** 1 Department of Clinical Psychology II, Institute of Psychology, University of Innsbruck, Innsbruck, Austria; 2 AMEOS Clinic for Psychiatry and Psychotherapy, Hildesheim, Germany; 3 Institutes for Forensic Medicine, Hannover Medical School, Hannover, Germany; 4 Department of Psychiatry, Social Psychiatry and Psychotherapy, Hannover Medical School, Hannover, Germany; 5 Center for Systems Neuroscience Hannover, Hannover Medical School, Hannover, Germany

**Keywords:** Predictive preventive personalized medicine (PPPM/3PM), Hair cortisol level, Major depressive disorder, Suicide, Risk monitoring, Biomarker pattern, CoViD-19 pandemic relevance

## Abstract

Depression and suicidal behavior are interrelated, stress-associated mental health conditions, each lacking biological verifiability. Concepts of predictive, preventive, and personalized medicine (3PM) are almost completely missing for both conditions but are of utmost importance. Prior research reported altered levels of the stress hormone cortisol in the scalp hair of depressed individuals, however, data on hair cortisol levels (HCL) for suicide completers (SC) are missing. Here, we aimed to identify differences in HCL between subject with depression (*n* = 20), SC (*n* = 45) and mentally stable control subjects (*n* = 12) to establish the usage of HCL as a new target for 3PM. HCL was measured in extracts of pulverized hair (1-cm and 3-cm hair segments) using ELISA. In 3-cm hair segments, an average increase in HCL for depressed patients (1.66 times higher; *p* = .011) and SC (5.46 times higher; *p* = 1.65 × 10^−5^) compared to that for controls was observed. Furthermore, the average HCL in SC was significantly increased compared to that in the depressed group (3.28 times higher; *p* = 1.4 × 10^−5^). A significant correlation between HCL in the 1-cm and the 3-cm hair segments, as well as a significant association between the severity of depressive symptoms and HCL (3-cm segment) was found. To conclude, findings of increased HCL in subjects with depression compared to that in controls were replicated and an additional increase in HCL was seen in SC in comparison to patients with depression. The usage of HCL for creating effective patient stratification and predictive approach followed by the targeted prevention and personalization of medical services needs to be validated in follow-up studies.

## Introduction

### An epidemiological perspective on depression and suicide

Suffering from chronic or traumatic mental distress is one of the most contributing risk factors for the etiology of mental disorders [1] including major depressive disorder (MDD). Although about 25% of all disability-adjusted life years lost in Europe are due to mental disorders [2], relatively little is known about the underlying pathophysiological mechanisms. As a consequence, mental disorders remain a strong socioeconomic burden to all societies [3] characterized by relatively poor treatment outcomes. The impact of this burden is highlighted by reports that up to 15% of the general population develop at least one depressive episode in a lifetime [1]. This emphasizes and underlines the need for strong scientific efforts to promote the identification of preventive, predictive, and personalized medical (3PM) treatment options in affective disorders including MDD as a paradigm shift from today’s reactive psychiatric services.

Epidemiological research identified risk factors for MDD, reporting age, sex, socioeconomic status, social support, and lifestyle factors as relevant variables [4]. MDD is not only associated with impaired life quality [5], but it is further associated with premature morbidity and mortality due to e.g. suicidal behavior [6, 7]. In Europe, the yearly loss of lives includes about 60,000 individuals who die by suicide. Even worse, for each completed suicide, twenty other non-fatal suicide attempts can be considered statistically [8]. This unacceptable high number of cases further accentuates the utmost urgent need to promote and establish the principles of 3PM (for example see [9–11] also in psychiatric healthcare (see [12] for pharmacogenomics in the treatment of mood disorders)), especially predictive and preventive strategies in those associated with suicidality. In contrast to the risk for MDD, both suicidal risk and fatal suicide attempts are more strongly associated with the male sex [13]. Additionally, men have a higher risk to perform the transition from suicide idealization to completing suicide [14]. Adolescent suicide has also been related to the impact of different sociocultural, psychological, psychopathological, and biological factors (for a review see [15]). A distinct link between psychiatric conditions and suicide risk has been reported: About 90% of all suicide completers (SC) are estimated to have a psychiatric disorder at the time point of death [16, 17]. Here, MDD contributes approximately 60% to the total number of deaths [18, 19]. Although suicidal behavior is not limited to MDD, it plays a very important role due to the highest risk of attempting or dying by suicide in this group of individuals [20]. The impact of suicide in combination with MDD is reflected in findings showing a worldwide rate of suicide of about 800,000 per year [8]. Again, this very high number of yearly life losses emphasizes also the need for better predictive and preventive strategies and 3PM implementation towards personalization of medical services in psychiatric services to significantly reduce the very high number of suicides around the globe. Another important aspect to be considered in this context is the ongoing CoViD-19 pandemic and its associated negative health complications [21] and outcomes on mental health [22] that can be linked to changes in suicide rates (for a review see [23]) with country-specific differences.

### Missing biomarker availability in depression and suicide

Depression and suicidal behavior are interrelated, stress-associated mental health conditions, each lacking biological verifiability. Missing biomarker availability for both conditions leads to negative medical outcomes of 3PM-related aspects in terms of treatment efficacy, remission stability, and relapse probability. For example, suicidal behavior and ideation contribute to the severity of MDD and worsen the therapeutical access and the efficacy of antidepressant treatment. As a consequence, about 25% of depressed individuals commit suicide while having been in contact with psychiatric services shortly before the time of their death [24, 25]. Here, predictive biomarkers for a psychobiological stress burden would help to prevent at least cases of severe depression and associated suicide risk. Known psychosocial risk factors for fatal suicide attempts include social and financial problems, traumatic life events, and the loss of a family member or friend [26]. However, a biologically embedded 3PM approach to identify and monitor physiological dysregulation of stress response mechanisms as a consequence of chronic or traumatic stress exposure in the context of MDD and suicide risk is most urgently needed, but still missing. Therefore, more interdisciplinary research on suicide risk and completed suicide is necessary to identify new biomarker candidates; the psychoneuroendocrine stress response has been intensively targeted as one potential starting point for this approach.

### Cortisol and the neuroendocrine stress response

As part of an acute response, the body’s neuroendocrine stress response is triggered by environmental stressors, resulting in the stimulation of the hypothalamic-pituitary-adrenocortical (HPA) axis to release the steroid hormone cortisol from the adrenal glands into the bloodstream. Under conditions of sufficient chronicity, amplitude, and/or frequency, a stressor can trigger a maladaptive regulation of the stress response system [27], resulting in alterations of the HPA-axis signaling and regulation as reflected in persistent changes in cortisol levels. From a more holistic perspective, the dysregulation of the neuroendocrine axis leads to (mal)adaptative changes also in other biological systems, including the central and the autonomous nervous systems, and the immune system. Therefore, stress-related pathological burden is discussed to affect the body on a systemic level, suspecting MDD as a disease that negatively affects the body’s functionality as a whole [28].

### Research findings on cortisol alterations in depression

Alterations of HPA signaling and resulting cortisol levels have been reported in many stress-related conditions. Studies investigating MDD showed hypercortisolism states [29, 30], but also opposite/mixed findings [31, 32] or even no differences to non-stressed control groups were found [33–35]. This inconsistency of observations can be related to differences in the selected biomaterial collected for analysis, differences in sample preparation, and technical and/or methodological differences used for quantitative steroid analysis. Towards achieving a technical and methodological standard, harmonization of protocols and analytical procedures is a prerequisite for the promotion of 3PM concepts in clinical psychology and psychiatry. Another explanation for the reported discrepancies might lie in demographic, socioeconomic, and lifestyle differences of participants including age, sex, gender, physical activity, nutrition, the chronicity and strength of psychological stress burden, and social support between the studies. From a biological perspective, the diurnal regulation of neuroendocrine cortisol release further can cause difficulties in standardizing the time point of sample collection in human research [36]. One methodological approach to overcome these limitations is represented by the usage of human scalp hair for the measurement of hair cortisol levels (HCL).

### Research on hair cortisol in chronic stress conditions

The assessment of cortisol in scalp hair allows the characterization of HPA-mediated stress responses in a retrospective manner over periods ranging from weeks to months depending on the length of hair strands used for HCL quantification. As an extension of conventional methodological approaches, hair cortisol overcomes the limitation of a snapshot impression of circulating cortisol concentrations in the periphery (e.g. blood, saliva) or cortisol secretion over a relatively short period (urine) of less than 1 day [37]. With a reported average hair growth of 1 cm per month [38–40], cortisol concentrations in 1-cm hair segments allow the retrospective assessment of average cortisol levels over approximately the past 4 weeks, and 3-cm hair strands over the past 3 months, respectively. Easy procedures of collection (non-invasive), storage (room temperature), and analytical handling (standardized operating procedures) of samples together with relatively high compliance of individuals and patients for sampling highlight hair as a very promising matrix towards 3PM approaches in psycho-bio-medical (stress) research. Among other areas of application, the usage of hair is already present in forensic, environmental, doping, and toxicology research [38–41], and most recently, there is increasing data also from stress-related and psychopathological stress research. Here, higher HCL have been reported e.g. in caregiving relatives of patients with dementia and chronically ill individuals [42], long-term unemployed vs. employed individuals [43], individuals with posttraumatic stress disorder (PTSD) [44], women with childhood maltreatment [45], and individuals with depression [46–50]. However, based on the relatively low number of studies, also mixed results and heterogeneous findings were reported for HCL in depressed patients. A systematic review and meta-analyses by Psarraki and colleagues [51] summarized the available literature and discussed strengths and limitations of the approach.

In comparison to the biology of MDD, today relatively little is known about the pathophysiological underpinnings of suicidal behavior and suicide attempts. But similar to MDD, literature provided evidence for an association between alterations in HPA regulation and suicide [52–55]. For example, higher levels of corticotropin-releasing hormone (CRH) and vasopressin found in the frontal lobe, the raphe nuclei, and the locus caeruleus of SC strengthened the perspective of a contributing role of HPA-axis functioning in suicide (for a review, see [16]). Furthermore, the observed decreased density of CRH receptor 1 in the frontal cortex may possibly be linked to an adaptive mechanism in response to increased release of CRH. On the level of gene expression, reduced levels of glucocorticoid receptor mRNA were found in the amygdala and the prefrontal cortex associated with suicide [56]. Altogether, these post-mortem findings are in line with results from animal studies using chronic stress models [57]. To the best of our knowledge, no data is available on HCL in SC. However, a possible (and easy obtainable) biomarker correlate of suicidal risk — with the additional possibility to discriminate between depressed and non-depression suicide attempts — would be an important 3PM-oriented predictive as well as preventive tool in psychiatric research. Consequently, it was aimed to test the usage of HCL as a correlate of stress-related HPA-axis (mal)adaptation first in the context of depression and depression severity, which might help to further characterize and better understand the endocrine consequences of MDD. Additionally, HCL may represent a powerful tool to track neuroendocrine changes in the process of psychotherapy-based interventions in future studies. Second, HCL was investigated in a group of SC to evaluate its usage as a possible indicator candidate for endocrine alteration reflecting an increased risk for committing suicide. In general, a possible biomarker correlate of suicidal risk — with the additional possibility to discriminate between depressed and non-depression suicide attempts — would be an important 3PM-oriented predictive as well as preventive tool in psychiatric research. It was hypothesized that HCL is elevated in both MDD and SC compared to that in a mentally stable, non-depressed group of age-matched controls. Furthermore, it was expected that in individuals who completed suicide, HCL further exceeds the level of that observed in individuals with MDD, which might contribute to the risk for the “final exit” behavior of committing suicide. In the present study, a first exploratory approach was conducted to characterize HCL in the context of depression and suicide to also stimulate more 3PM-oriented investigations in this very important but rather neglected field of bio-psycho-medical research.

## Methods

### Recruitment and clinical characterization of non-depressed controls and depressed patients

Mentally stable females (*n* = 22) without any history of depression or any other mental disorders (control group; CG) were recruited via public advertisement using posters and local newspaper announcements in Hildesheim, Germany. Women with a current diagnosis of MDD (*n* = 20) according to DSM-IV [58] were inpatients at the AMEOS Clinic for Psychiatry and Psychotherapy in Hildesheim. Besides the characterization of immunocellular and biomolecular alterations in depression [59–61], study participation also included the collection of hair samples voluntarily. Both groups were screened for MDD by trained psychiatrists using the *Structural Clinical Interview* (SCID-I). Further, the questionnaires *Beck’s depression inventory-II* (BDI, self-report; [62]) and the *Montgomery-Asberg Depression Rating Scale* (MADRS, interview; [63]) were used to assess the current severity of depressive symptoms. Exclusion criteria for both groups contained the presence of neuropsychiatric comorbidities including *Parkinson’s* and *Alzheimer’s* disease, schizophrenia, eating disorders, and other clinically relevant neuropsychiatric conditions. To control for known confounders on HCL, body mass index (BMI), current tobacco consumption, and the level of physical activity were additionally assessed for the CG and the group of patients with MDD.

### Collection of human scalp hair

Hair samples from control subjects (*n* = 14) and depressed patients (*n* = 20) were collected at the AMEOS Clinic for Psychiatry and Psychotherapy in Hildesheim. Hair samples from SC (*n* = 45) were collected at the Institutes for Forensic Medicine at the Hannover Medical School. As part of the clinical routine procedure, a distinct amount of hair from all SC with unknown death backgrounds is collected and tested to exclude intoxication or other chemical influences highlighting the possibility of third-party death responsibility. The remaining hair samples collected for this purpose were used in this study for the analysis of HCL. Due to data protection reasons and ethical restrictions, no information on current medication intake and clinical history of somatic or psychiatric diseases was accessible; however, information regarding age, BMI, and sex was made available. Data on BMI was missing for *n* = 9 subjects of the SC group. Hair strands from SC possibly contaminated with blood, urine, or other biological fluids during the visual inspection of the body were excluded from sampling. Two samples from the control group were excluded from the analysis due to reported chemical treatment (bleaching), resulting in a total of *n* = 12 samples for the control group. Demographic, as well as clinical, characteristics of the control group and the depressed group are provided in Table 1.

**Table 1:** Mean and *SD* of demographic and clinical characteristics of the study cohort (non-depressed controls (CG, *n* = 12), depressed patients (MDD, *n* = 20), and suicide completers (SC, *n* = 45))

Variables	Group			*U-value*	*Z-value*	*Chi*^*2*^	*p-value*
	CG (*n* = 12)	MDD (*n* = 20)	SC (*n* = 45)				
DSM-IV							
Mild (*n*, (%))	0	4 (20)	NA				
Moderate (*n*, (%))	0	6 (30)	NA				
Severe (*n*, (%))	0	10 (50)	NA				
CGI score (mean ± *SD*)	1.92 ± .29	5.2 ± 1	NA				
BDI score (mean ± *SD*)	2.25 ± 1.77	24.85 ± 10.51	NA	0	− 4.68		**3 × 10**^**−5**^
MADRS score (mean ± *SD*)	1.67 ± 2.1	24.5 ± 8.55	NA	0.5	− 4.69		**3 × 10**^**−5**^
Cortisol in hair, 3-cm segment (ng/ml)	.075 ± .022	.126 ± 0.069	.412 ± .478			34.8	**2.77 × 10**^**−8**^
Cortisol in hair, 1-cm segment (ng/ml)	.071 ± .019 (*n* = 5)	.111 ± .0396 (*n* = 7)	.136 ± .123 (*n* = 10)				.427
Chronological age (mean ± *SD* years)	57 ± 5	58.5 ± 6.3	55.09 ± 16.82			.624	.73
Sex (*n*, (females (%))	12 (100)	20 (100)	17 (37.8)			31.29	**1.6 × 10**^**−7**^
BMI (mean ± *SD* kg/m^2^)	23.95 ± 3.1	28.62 ± 7.55	25.31 ± 5.21 (*n* = 36)			4.42	.11
Current smoker (*n*, (yes in %))	4 (18.2)	10 (50)	NA			.847	.36
Physical activity (*n*, (yes in %))	10 (83.33)	13 (65)	NA			1.247	.26
Documented medication							
Antidepressants (*n*, (yes in %)	0	16 (80)	NA				
Antipsychotics (*n*, (yes in %)	0	9 (40.9)	NA				
Hair color							
Black (*n*, (%))	0	1 (5)	8				
Brown (*n*, (%))	9 (75)	15 (75)	27				
Blond (*n*, (%))	0	1 (5)	4			15.42	.35
Grey (*n*, (%))	2 (16.66)	3 (15)	3				
White (*n*, (%))	0	0	2				
Red (*n*, (%))	1 (8.34)	0	0				

*U*-value and *Z*-value are listed for two-tailed Mann–Whitney *U* test, and Chi^2^ for Pearson’s chi-squared test. Bold *p*-values indicate significance on an alpha level of .05

Abbreviations: *SD*, standard deviation; *DSM-IV*, Diagnostic and Statistical Manual for Mental Disorders IV-TR; *BDI*, *Beck depression inventory*-II; *MADRS*, *Montgomery-Asberg depression rating scale*; *BMI*, body mass index; *NA*, not available

### Processing of scalp-hair samples and quantitative cortisol assessment

Hair strands with a minimum diameter of 3 mm were cut as best as possible most proximal to the scalp skin avoiding the hair follicle. To exclude any contamination of the hair samples by direct skin contact, surgical gloves and ethanol (70%)–cleaned dry scissors were used to collect hair and to cover samples with aluminum foil. Hair samples were stored at − 20 °C until analysis. For the pre-analytical processing and isopropanol washing of samples, we performed the protocol given by Davenport and colleagues (2006) [64]. After the completion of the sample sets, all hair strands were first washed and then cut into 3-cm-long segments from the proximal cutting edge as it was primarily intended to investigate the cumulative secretion of cortisol within the past 3 months and human scalp hair grows approximately 1 cm per month [38]. The remaining hair was used for an additional 1-cm strand sample (proximal to the scalp, representing the last month before collection) using the same technical processing: In total, 50 mg of 3-cm hair segments (10 mg for the 1-cm segments, respectively) was placed into 2-ml tubes filled with sterile 1/8″ stainless steel beads processed in a FastPrep ball mill (MP Biomedicals, Germany) for 3 × 5 min at 3 Hz to pulverize the samples. From each sample, 25 mg of homogenized powered hair was translocated into a new sterile reaction tube for overnight extraction of cortisol in 1 ml methanol (HPLC-certified HiPerSolv Chromanorm, VWR International, Germany). Samples were centrifuged at 18,800 g (Fresco 21, Thermo Fisher Scientific, Germany) for 30 s at room temperature, and a supernatant volume of 600 µl was translocated in a new tube and dried in a CentriVap concentrator (Labconco, USA). The dry extracts were suspended in 220 µl sterile phosphate-buffered saline (PBS, Invitrogen, USA). Enzyme-linked immunosorbent assays (Saliva Cortisol kit, IBL, Germany) were performed with 50 µl of each sample — measured in duplicate — to assess cortisol following the manufacturer’s instructions. Samples were allocated and measured on the 96-well plate of the kit in randomized order, and given cortisol levels are averaged values of the duplicates of the same sample. The optical density of the sample wells was measured at 450 nm using an ELISA plate reader (TECAN M200, Germany). The assay kit provides a cortisol detection range from 0.015 to 3 ng/ml, with an analytical sensitivity of 0.003 ng/ml and a functional sensitivity of 0.005 ng/ml.

### Statistical analyses

Statistical analyses were performed using SPSS (IBM Corporation, USA). Data were tested for normal distribution using *Kolmogorov–Smirnov* tests, and non-parametric group comparisons were performed using the *Kruskal–Wallis H* test when conditions for parametric ANOVA were not fulfilled. Statistical analyses based on the hypothesized expectation of a positive association between depressive symptoms and HCL were performed using one-tailed tests. Post hoc corrections for multiple comparisons were performed with Bonferroni. For non-parametric groupwise comparisons, *Mann–Whitney U* tests were selected, while parametric testing was performed using *Fisher’s t*-test. Pearson’s *χ*^2^ tests were applied for categorical, dichotomous variables. Non-parametric correlation analyses were performed using Kendall’s *τ*. Residuals were tested for normality and equality of variance. The level of significance was set to *p* = 0.05. *Kolmogorov–Smirnov* tests revealed that age, hair cortisol levels in 1-cm and 3-cm hair strands, sum scores of both BDI and MADRS, and BMI were non-normally distributed (all *p* values ≤ 0.05). The three groups did not significantly differ for age (*χ*^2^ = 0.624, *df* = 2, *p* = 0.732) and BMI (*χ*^2^ = 4.422, *df* = 2, *p* = 0.110). The comparison between the CG group and the MDD group showed no significant difference in the percentage of cigarette smokers (*χ*^2^ = 0.847, *df* = 1, *p* = 0.732) or physical activity patterns (*χ*^2^ = 0.264, *df* = 1, *p* = 0.264). As expected, patients with MDD scored significantly higher in the clinical instruments BDI-II (*U* = 0, *Z* =  − 4.678, *p* = 3 × 10^−6^) and MADRS (*U* = 0.5, *Z* =  − 4.691, *p* = 3 × 10^−6^) compared to the control group.

## Results

### Between-group comparison of hair cortisol levels 

**Fig. 1:**
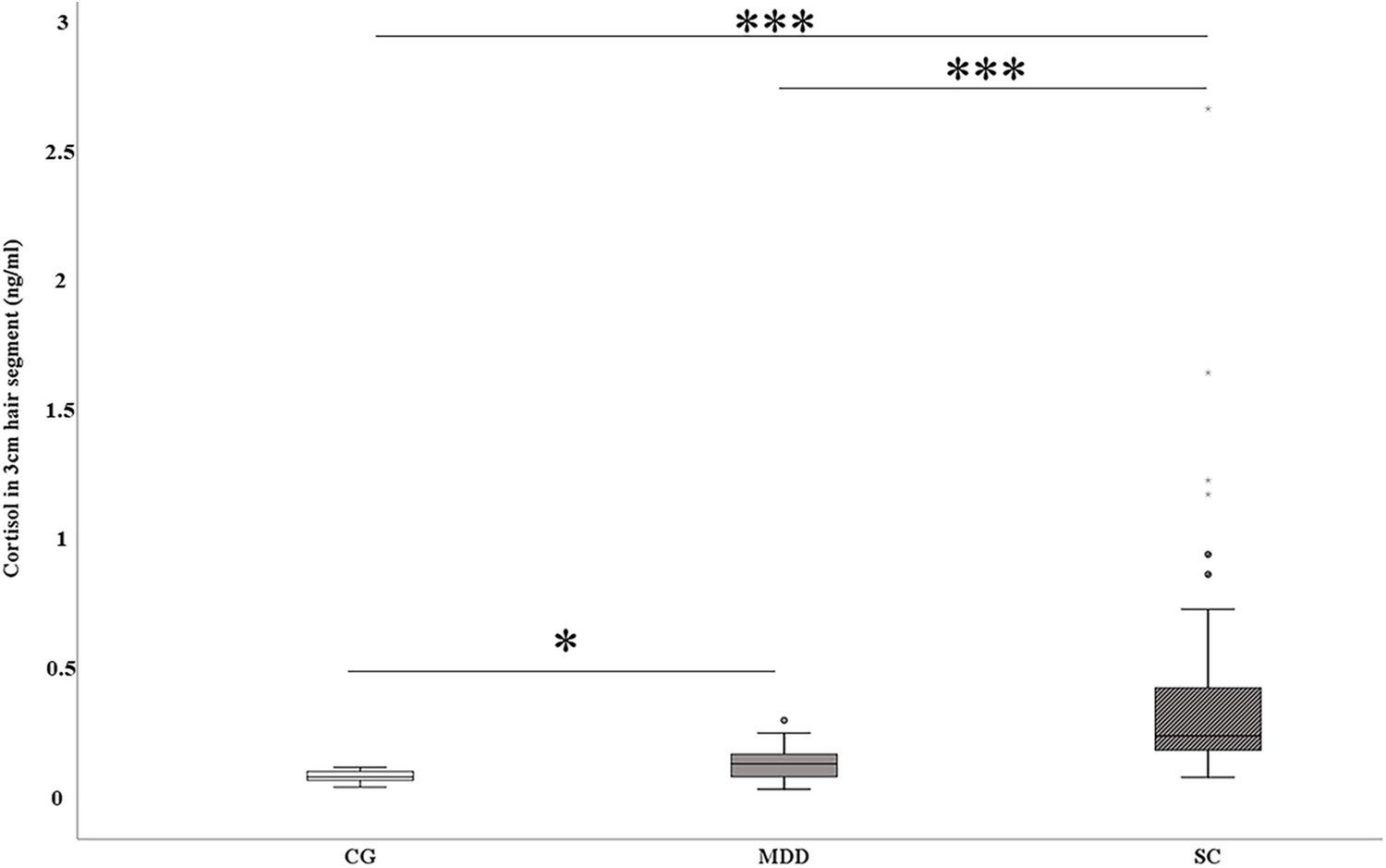
Comparison of cortisol levels (ng/ml) in 3-cm hair segments between non-depressed controls (CG), depressed patients (MDD), and suicide completers (SC). Asterisks mark significance levels: *p < .0166; ***p < .00001. *p* values were adjusted with *Bonferroni* for alpha error correction due to multiple testing (*p*_adj_ = .0166). Asterisks and dots within the SC group mark male subjects with the top six cortisol values

When comparing the 3-cm hair strands, non-parametric *Kruskal–Wallis* testing revealed a significant difference in HCL between the three groups (*χ*^2^ = 34.8, *df* = *2*, *p* = 2.77 × 10^−8^). The groupwise comparison applying *Mann–Whitney* between the control group and the MDD group showed a significant difference with higher HCL in the depressed group (*U* = 61, *Z* =  − 2.297, *p* = 0.011 [one-tailed]). Also, the difference between the CG and the SC group was statistically significant (*U* = 82, *Z* =  − 4,152, *p* = 1.65 × 10^−5^ [one-tailed]) as well as the difference between MDD and SC (*U* = 155, *Z* =  − 4.193, *p* = 1.4 × 10^−5^ [one-tailed]). All significant *p* values remained significant after Bonferroni correction (*p*_adj_ = 0.0166) for multiple testing. A graphical representation of the group comparison is given in Fig. 1.

### Correlation between the severity of depressive symptoms and HCL

**Fig. 2:**
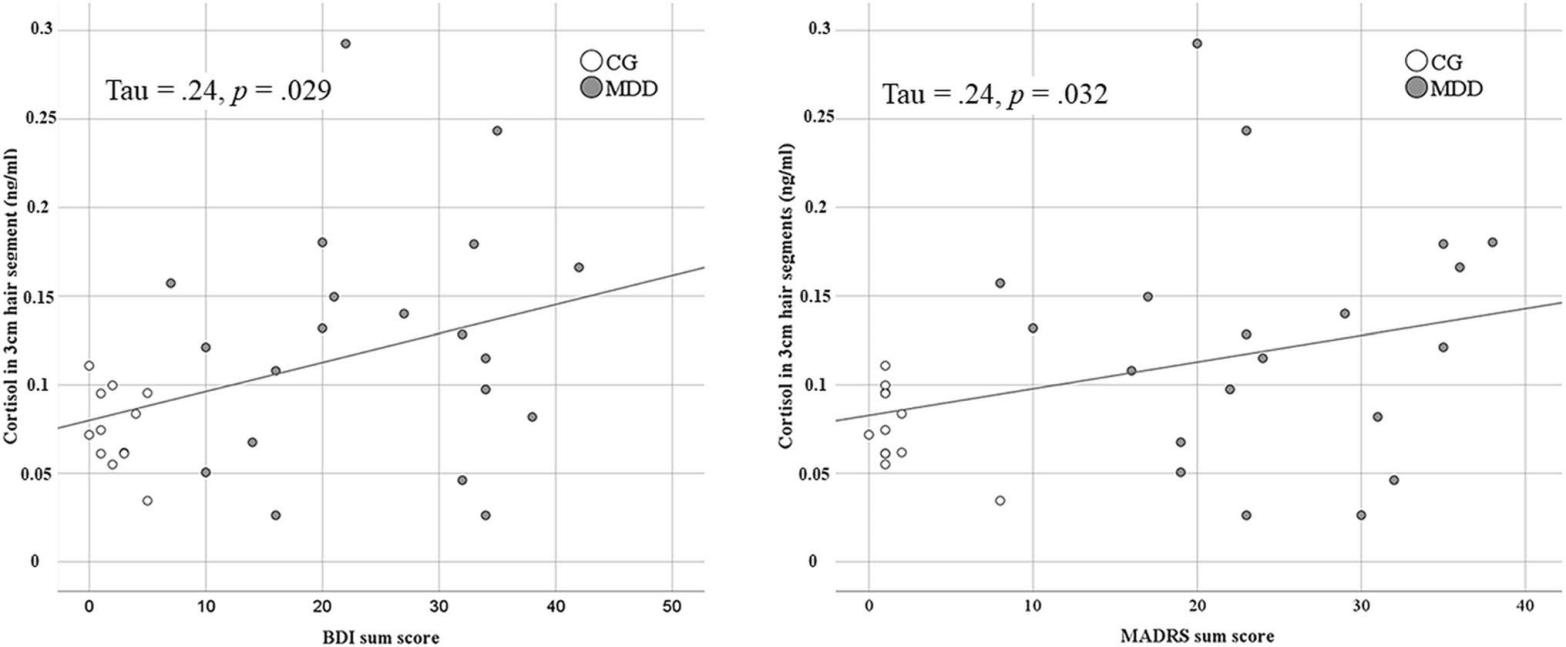
Graphical representation of the correlation analysis between the severity of depressive symptoms, assessed with the BDI sum score (left, self-report questionnaire) and the MADRS sum score (right, interview) and the cortisol levels in 3-cm hair segments. CG: Control group, MDD: Depressed patients

Using Kendall’s *tau*, the correlation analysis between the clinical severity of depressive symptoms, represented in the sum scores of the clinical self-report questionnaire BDI, and cortisol levels in the 3-cm hair strands showed a significant correlation between the two variables (*tau* = 0.240, *p* = 0.029 [one-tailed]). Also the MADRS sum scores (interview-based ratings) were tested for an association with HCL in the 3-cm hair segments and a significant correlation (*tau* = 0.237, *p* = 0.032 [one-tailed]) was found. A graphical representation of the correlation analyses is given in Fig. 2.

**Fig. 3:**
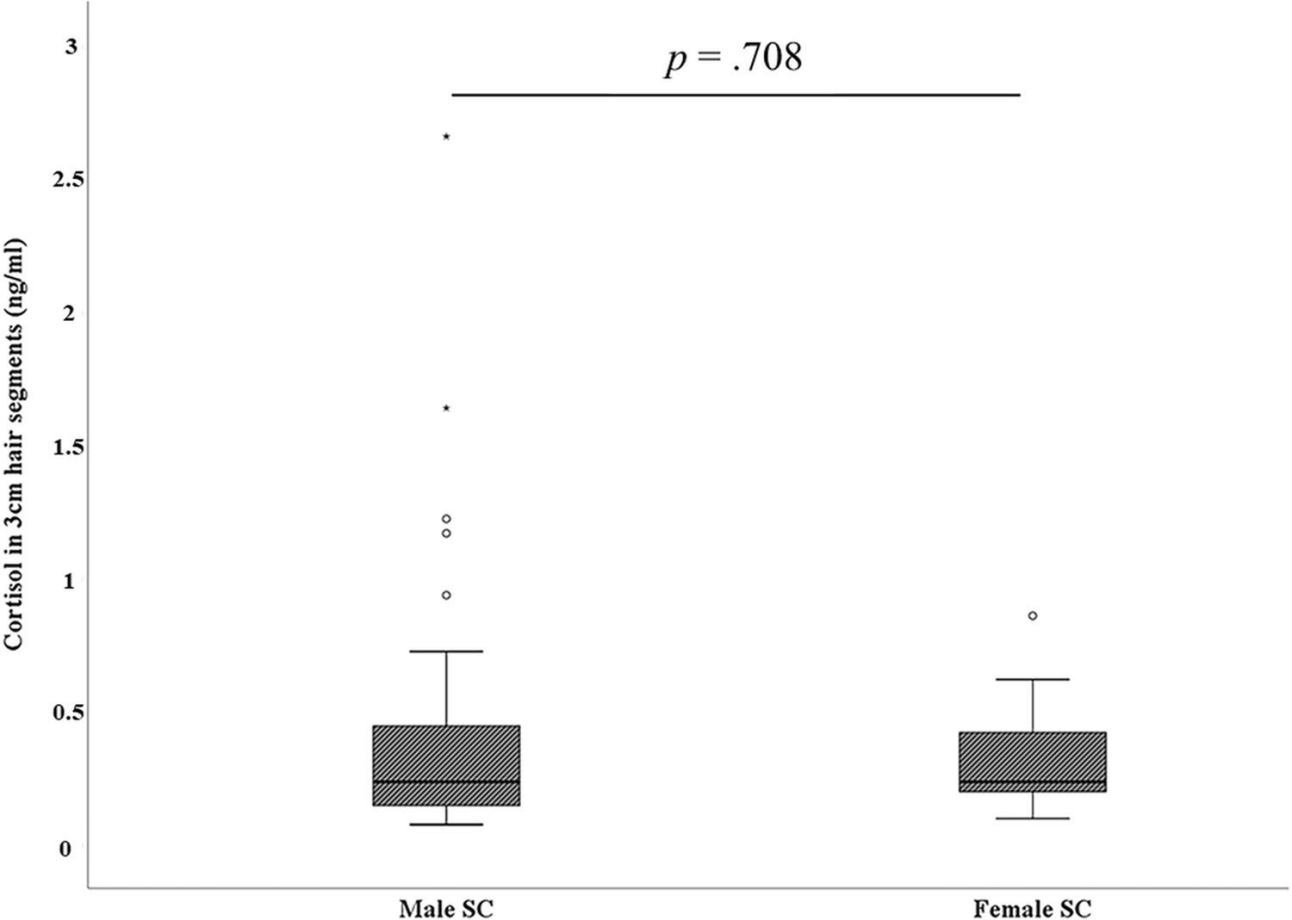
Comparison of hair cortisol levels in 3-cm hair segments between male and female suicide completers (SC)

To control for known confounding variables on HCL, a general linear model with hair cortisol values and BDI sum score was used that did not show a significant effect neither for age (*df* = 1, *F* = 0.455, *p* = 0.515) nor for BMI (*df* = 1, *F* = 2.807, *p* = 0.125) used as covariates in the analysis. The same approach was performed for the MADRS sum score, where again neither age (*df* = 1, *F* = 0.426, *p* = 0.527) nor BMI (*df* = 1, *F* = 0.003, *p* = 0.956) showed a significant effect on the association between HCL and the severity of depressive symptoms. Finally, it was investigated whether sex had an effect on HCL within the group of SC. Although men showed higher mean HCL (Fig. 3), no significant differences between the sexes were observed (*U* = 222, *Z* =  − 0.375, *p* = 0.708).

### Comparison of HCL in 3-cm and 1-cm hair segments

**Fig. 4:**
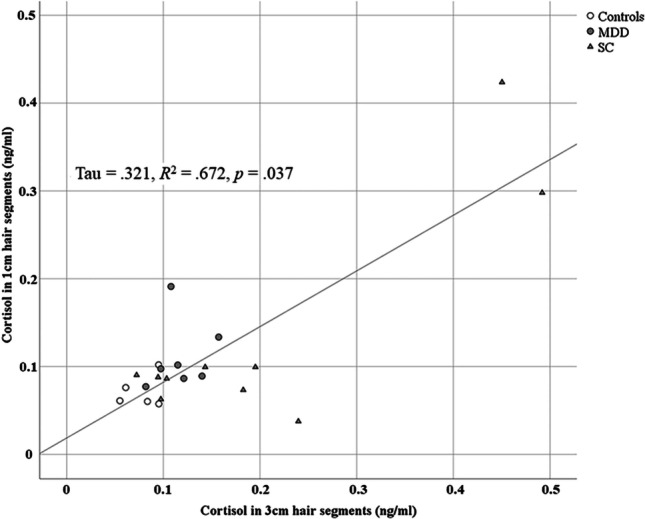
Kendall’s *τ* correlation between the hair cortisol level in 1-cm and 3-cm hair strand segments including the control group (CG), depressed patients (MDD), and suicide completers (SC)

To elaborate more insights into the time-related dynamics of HCL alterations in MDD and SC, cortisol data from the 3-cm hair strand segments was compared with data of a subset of samples that provided sufficient hair material to additionally measure HCL in the 1-cm segment. In total, hair cortisol data from *n* = 5 control subjects, *n* = 7 depressed patients, and *n* = 10 subjects from the SC group was included into the analysis. Non-parametric correlation analysis using *Kendall’s tau* revealed a significant and positive correlation (*tau* = 0.321, *R*^2^ = 0.672, *p* = 0.037) between the two hair segment lengths available from the same donors (Fig. 4).

## Discussion

The primary aim of this study was to identify and characterize possible differences in HCL between depressed patients and SC, which were compared to a group of non-depressed control subjects. First, a higher average HCL in the hair of depressed patients was found compared to that in the controls. This result is in line with previous findings showing long-term elevated HCL as supporting evidence for changes in HPA-axis regulation and neuroendocrine signaling via cortisol secretion in MDD: The presented findings replicate and support previous reports of hypercortisolism in MDD (at least) in hair [46, 47, 49, 50]. However, it should be noted that in general hair data cannot be directly compared to study results on cortisol levels measured in other biofluid materials (blood and/or saliva). More research on the comparability of cortisol levels in hair with other biological matrices is therefore necessary to better understand the physiological condition of increased HCL on a systemic level. Additionally, a significant correlation was found between the clinical severity of depressive symptoms and HCL, suggesting a dose-dependent effect of depression-associated stress on HCL.

To our best knowledge, this is the first study to show not only an association between psychological (severity of depressive symptoms) and biological (hair cortisol levels) changes in MDD but also that this association can be expected independently from the clinical instrument (BDI vs. MADRS) used to assess the severity of depressive symptoms. In line with the 3PM concept, it could be argued HCL may be used not only as a retrospective marker of psychobiological stress load, but also as a prognostic marker of clinical remission after therapeutical intervention. More intensified research has to be initiated in the context of preventive, predictive, and personalized medicine with longitudinal study designs to further elaborate the potential of HCL as a translational approach to clinical routine.

Interestingly, a significant positive correlation was found between HCL measured in the 1-cm and the 3-cm hair segments. Here, it was possible to provide first data showing the potential of hair cortisol analysis. In contrast to the control group, depressed patients with depression showed chronically elevated cortisol levels, contributing to endocrine evidence that depression is associated with chronic psychobiological stress. This further strengthens the perspective that both MDD and suicide are two conditions associated with the exposure to chronic and/or traumatic distress, while inter-individual alterations could also be observed in our results (see Fig. 4).

In the given work, the very first data on cortisol alterations measured in the hair of individuals who completed suicide are presented. This is of special interest because our analyses revealed a significant difference in HCL between SC and controls and also between SC and individuals suffering from depression. Noteworthy, no significant difference in HCL between male and female SC was observed. Therefore, neuroendocrine mechanisms associated with suicide do not seem to show sex specificity. The presented findings of hypercortisolism in the groups hypothesized to be associated with chronic stress are contrary to results from other stress-related conditions with reported alterations in HPA-axis regulation, including hypocortisolism in PTSD [44] and also depression [32], or even no changes in (female) patients with borderline personality disorder [65]. The precise character of this possible physiological difference between these clinical cohorts needs to be further elaborated. The identified group differences in HCL between MDD and SC provide arguments to expect that the neuroendocrine alterations in the context of suicide even exceed the cortisol-associated pathophysiology of MDD. As summarized by Miret and colleagues (2013) [66], there are unmet needs in psychiatric research focusing on the global problem of suicidal behavior. The results support the expectation that hair cortisol measurements hold the potential to become a relevant, robust, and easily applicable tool to estimate and monitor the psychobiological stress load and its concurrent pathophysiological relevance in terms of 3PM in clinical psychology and psychiatry. As high distress is a prevalent phenomenon in individuals as well as in many societies around the globe, improvements in preventive and predictive strategies, e.g. with the use of HCL for the physiological evaluation of neuroendocrine risk and resilience, and for monitoring and suppressing health-to-disease transition rates, would significantly contribute to a lowering of both depression and suicide rates. The urgent need to improve the monitoring possibilities for practitioners is represented in epidemiological data showing that primary care plays a very important role in suicide prevention. For example, individuals who have died by suicide had seen a general practitioner in the 3 months before death, 40% in the month forehand, and 20% in the week before death [67, 68]. Used as a biomarker for psychobiological stress load, HCL might provide an indicator for the potential risk of suicide attempts. Future research has to test the concept and the robustness of the first findings presented here, at best using longitudinal study designs including treatment interventions.

On a physiological level, exposure to chronic stress has not only been associated with an increased neuroendocrine cortisol response, but further acts on other biological stress response systems including the immune system. Long-term exposure to elevated cortisol levels can negatively affect immune reactivity, auto-immune regulation, and immunity due to its immunosuppressive function. In addition, impaired cortisol signaling, e.g. as a result of glucocorticoid resistance at the receptor level, can result in elevated inflammation. Increased inflammatory signaling — as reflected in higher levels of proinflammatory cytokines and elevated cell numbers of innate as well as adaptive immune cells — has been reported consistently to play a central role in the pathophysiology of MDD (for a review, see [69]). This perspective of negative health outcomes associated with chronic stress exposure in depressed patients is strengthened by other biological findings, e.g. shorter telomeres in peripheral blood immune cells [70], changes in immune cell bioenergetics [59], and increased levels of free-circulating mitochondrial DNA (mtDNA) in blood [71], indicating allostatic load promoting processes of reduced immunocellular functioning, proinflammatory states, and premature ageing in MDD. Besides findings demonstrating changes in mtDNA serum levels in suicide [72], data related to telomere length dynamics in individuals with suicidal tendencies or even after fatal suicide attempts are missing. However, the endocrine changes reported in subjects with suicidal behavior raise the expectation that neuroendocrine alteration also affects the function of other stress regulatory systems. Therefore, it can be expected that not only inflammatory processes but also cognitive alterations occur in subjects with suicidal behavior. The latter can be assumed especially by the accepted finding of hippocampal sensitivity towards chronic exposure to cortisol (for a review, see [73]). But also other cognitive functions seem to be impaired in both conditions: For example, electrophysiological evidence from electroencephalography (EEG) studies showed changes in the evaluation of environmental conflicts and errors in both depressed patients and suicide attempters, reflected in the *error-related negativity* (ERN), an event-related potential observable after the direct and indirect exposure to conflict-loaded stimuli [74]. One possible explanation might be that elevated cortisol levels — the result of prolonged stress exposure — trigger the individual to behavioral adaptation with the primary goal to increase the individual’s probability of survival. As a consequence, possible environmental or cognitive conflicts need to be suppressed on the behavioral level by decreasing the conflict monitoring–related communication between the limbic anterior-cingulate cortex (ACC) and the prefrontal cortex. However, the chronicity, duration, and intensity of cortisol signaling might show a dose–response effect in this crosstalk. One might speculate that the difference between non-suicidal depressed and (depressed) SC might lie in the way conflicts are processed and transduced into a cognitive adaptation towards prolonged stress exposure. While depressed individuals are still able to realize the resulting conflict of suicidal tendencies as a possible life threat, preventing them to overcome the threshold from depression to suicide, suicide attempters lack this conflict-monitoring capability, resulting in a suicide attempt becoming more probable. This functioning of the cognitive evaluation of conflicts might contribute to the decision whether individuals conduct suicide attempts or not. Finally, by highlighting the need for more interdisciplinary research, future studies should address the aspect of ERN research in the context of depression, suicidal behavior, and suicide prevention in combination with the assessment of HCL in studies at best with a longitudinal design. In sum, these approaches would provide a continuously strengthening argument for interdisciplinary research towards 3PM.

## Limitations

Besides strengths, this study also includes limitations that need to be considered. One limitation is related to the exclusive recruitment of women in the control group and the MDD group, while samples from both sexes were obtained from the SC group. It is not possible to hypothesize whether findings of increased HCL can be extended to male controls and male patients with MDD. Nevertheless, increased HCL were found for male SC compared to females (see Fig. 3), but the mean HCL difference between the two groups was not statistically significant. Increased HCL were reported in the literature for men, young children, and older adults when compared to women, adolescents, and younger adults, respectively [75]. Future studies should, therefore, include males and also younger cohorts of non-depressed controls and subjects with depression to address this limitation. At best, also hair samples from individuals who died of non-suicide should be included. The identified alterations in HCL do not allow any speculation on cortisol alterations detectable in other biomaterials (e.g. saliva, urine, blood). To better understand its temporal and spatial distribution in the body, future research should assess cortisol in at least two different biomaterials. Here, it is worth noting that cortisol can also be detected in recently reported biomaterials including fingernails (for a review, see [76]) and cerumen [77].

Main hair characteristics (e.g. washes per week, hair formation, coloration) were not available except for visible evidence of bleaching, and therefore, we cannot conclude that these variables might not have an influence on HCL in the samples. Nevertheless, previous research found no significant impact by hair type, color, and color treatment of hair [75]. Additionally, current smoking levels or uses of contraceptives were also reported to not affect cortisol levels in hair [75]. However, the robustness of these findings has to be replicated by future research. In a meta-analysis, Hawton and colleagues [78] reported risk factors significantly associated with suicide. These factors included the male sex, a family history of psychiatric disorder, the severity of depression, hopelessness, and comorbid disorders including anxiety and misuse of alcohol and drugs. In this study, higher cortisol levels were found for male SC, but the difference compared to female SC was not statistically significant. Due to data protection and other ethical reasons, access to information regarding the other given variables was not available.

## Outlook

It is necessary to stimulate more research in this highly relevant field, highlighting the main limitations of the present work to improve the process towards a better understanding of psychosocial and biological factors in individuals with suicidal behavior. Suicidal behavior is not limited to MDD but can affect also individuals with e.g. bipolar disorders, schizophrenia, and other psychopathologies. As access to information regarding the psychopathological background of SC was not available, future research should consider the impact of a lifetime to current psychiatric diagnosis or chronic and traumatic stressors (e.g. childhood maltreatment or traumatic life events) in the context of cortisol measurement in hair from psychiatric patients. Another aspect of importance is related to the possible effects of antidepressant (AD) medication. The precise function of AD medication on HPA activity is not understood in full detail, as it needs further clarification of whether AD medication directly affects cortisol levels, or whether HPA changes might be secondary outcomes of stress-diminishing effects due to antidepressant pharmacological treatment. One biological approach to investigate the role of AD medication on treatment effects might involve the function of cortisol as a molecular regulator for a variety of biological pathways, e.g. the *nuclear factor k-light-chain-enhancer of activated B cells* (NF-kappa B), an important regulator of cell–cell signaling (regulation of cortisol receptor NR3C1 and apoptosis via the p53 pathway) [79]. Here, animal studies could help to better understand the biological alterations induced by AD medication, not only on the endocrine but also on a systemic level.

## Conclusions and expert recommendations in the framework of 3PM medicine

### The contribution of the current findings

The study results show that HCL is higher in patients with depression in comparison to mentally stable and non-depressed control subjects and that this increase is significantly correlated with the severity of depressive symptoms, assessed by interview as well as self-report. Additionally, HCL was found even more increased in SC compared to depressed subjects, a first observation that requires replication and verification by future research. Based on this initial finding, it can be supposed that chronic psychological stress leads to a long-term dysregulation of the endocrine stress axis and associated regulatory systems, increasing the risk for MDD and suicide in a dose-dependent manner. Experts are now invited to test the usage of HCL as a retrospective marker of stress-associated depression and a prospective marker of remission stability using longitudinal study designs.

### Implications and recommendations for personalized medicine, targeted prevention, and predictive diagnostics

Differences in hair cortisol between depressed individuals and suicide victims suggest that HCL might contribute to new diagnostic tools and therapeutic monitoring approaches and may also help to improve preventive strategies in clinical application by providing new access to biological monitoring of stress modalities in depression and suicidal behavior. Experts (e.g. general practitioners, psychiatrists, and clinical psychologists) now have to investigate whether HCL in suicide attempters can be used as an indicator of stabilization and clinical improvement. Finally, the implementation of predictive, preventive, and personalized medicine in the scientific field of clinical psychology and psychiatry holds a very strong potential for a highly effective and strong improvement in the medical support of individuals suffering from mental distress. In contrast to biofluids, HCL and other solid matrices (nail, cerumen) extend the portfolio of biomaterial available for medical examinations, with the advantage of uncomplicated and non-invasive collection, handling, and storage until laboratory analyses. This allows non-biologically experienced medical staff (e.g. resident psychologists and psychotherapists) to also gain access to 3PM approaches, resulting in a high catalytic effect towards a better, more tailored medical support of those affected by severe types of stress.

## Data Availability

The dataset generated in the present study is available from the corresponding author on reasonable request.
